# Mediating roles of perceived social support and sense of security in the relationship between negative life events and life satisfaction among left-behind children: A cross-sectional study

**DOI:** 10.3389/fpsyg.2022.1100677

**Published:** 2023-01-13

**Authors:** Na Liu, Xinzheng Li, Xuemei Ding, Haixia Liu, Xiaoli Zhang

**Affiliations:** ^1^School of Nursing, Binzhou Medical University, Yantai, China; ^2^School of Public Health and Administration, Binzhou Medical University, Yantai, China

**Keywords:** rural left-behind children, life satisfaction, negative life events, perceived social support, sense of security

## Abstract

**Objective:**

Life satisfaction is closely related to the quality of life. Previous studies showed that left-behind children have low life satisfaction levels due to their separation from their parents. Therefore, it is significant to explore the risk factors and protective factors of left-behind children’s life satisfaction to improve their life quality. Therefore, the purpose of this study was to determine the mediating roles of perceived social support and sense of security in the relationship between negative life events and life satisfaction among left-behind children.

**Methods:**

A survey was conducted on 281 left-behind children in rural Shandong, China using negative life events, perceived social support, a sense of security, and life satisfaction scales.

**Results:**

Negative life events had a negative impact on life satisfaction (*β* = −0.34, *p* = 0.001). In addition, not only does social support mediate between negative life events and life satisfaction (*β* = −0.21, *p* = 0.001), but also in between, security (*β* = −0.05, *p* = 0.030). Moreover, social support and security act as a chain intermediary between negative life events and life satisfaction (*β* = −0.03, *p* = 0.010), with an indirect effect share of 4.76%.

**Conclusion:**

Negative life events could directly or indirectly affect the life satisfaction of left-behind children through the chain-mediating effects of perceived social support or the sense of security alone. Perceived social support and the sense of security are two important targets for further improving the life satisfaction of LBC.

## 1. Introduction

Left-behind children (LBC) are children who are stayed at their hometown because both of their parents are working or one is working outside the hometown and the other cannot care for them (State Council of the People's Republic of China, 2016; Chang and Lu, 2018, p. 59; Tang et al., 2019). With the rapid development of the economy and acceleration of urbanization in China, increasing numbers of young and middle-aged laborers are choosing to leave the countryside to live and work in cities (Fellmeth et al., 2018). However, due to housing, educational and medical issues, et.al, most of the children of these migrant workers are left behind in their hometowns under the care of their grandparents, relatives or other temporary guardians (Qin et al., 2022). According to the Ministry of Civil Affairs of the People’s Republic of China statistics, there were 6.436 million LBC in rural areas as of the end of 2019 (Ministry of Civil Affairs of the People’s Republic of China, 2016). Long-term separation from their parents, inadequate care from temporary guardians, and poor living environment result in negative emotions and behavioral and psychological problems to a certain extent (Chang and Lu, 2018; Song et al., 2018; Akezhuoli et al., 2022), which will produce low life satisfaction among rural LBC. The life satisfaction of LBC is positively correlated with their quality of life and sense of well-being (Ye et al., 2020). The reduced quality of life and sense of well-being is not conducive to the growth of LBC (Zhao et al., 2018; Chai et al., 2019). Therefore, it is highly desirable to identify the influencing factors and mechanisms of life satisfaction among rural LBC in order to improve their quality of life. Previous studies have shown that negative the life events could directly affect life satisfaction of LBC (Angarne-Lindberg and Wadsby, 2009; Furniss et al., 2009; Guang et al., 2017; Zhang et al., 2021). But these studies did not reveal the underlying mechanism between social support and security.

Negative life events could cause individuals to have adverse emotional experiences and harm their physical and mental health. Stress theory suggests that negative life events can be sources of stress, with life satisfaction as the response, and other mediating factors (e.g., coping style and emotional regulation) playing mediating roles between stress and its response (Peirce et al., 1996). A recent study empirically demonstrated the role of core self-evaluation in mediating the relationship between negative life events and life satisfaction among college students (Junjun and Zhenzhen, 2018). However, most researches on LBC have only find that negative life events were negative related with life satisfaction (Guang et al., 2017; Zhou et al., 2020; Zhang et al., 2021). In order to explore more protective factors to reduce the impact of negative life events on the life satisfaction of left-behind children. The research aimed at identifying that the role of social support and sense of security between life events and life satisfaction among LBC in rural China.

Some studies have found that the negative life events encountered by LBC were mostly conflicts in the processes of getting along with family, friends, or teachers, which made LBC feel unworthy of being loved and thus unwilling to seek external support and help (Szcześniak and Tułecka, 2020; Lu et al., 2021). Perceived social support refers to the subjective evaluation by an individual of the support they receive from outside sources (family, friends, or other interpersonal relationships; Wawrzynski et al., 2021). Rodríguez-Cano et al. found that perceived social support was an important factor of life satisfaction and influenced subjective perceptions the life status. The psychological state theory also suggests that life satisfaction is correlated with individual perceptions. It means that when people perceive more social support (Gönültaş et al., 2020), they will feel more care, attention and help from the outside world (Schwartz et al., 2010). Meanwhile, they will have a stronger sense of belonging and hope. Decreased perceived social support leads to decreased life satisfaction, which is associated with negative psychological states such as loneliness, depression and anxiety (Reid et al., 2022; Zhang and Dong, 2022). When encountering life events, LBC are unable to get the benefits of social support and ultimately their lives become uncomfortable and unpleasant, thus reducing their life satisfaction (Aune et al., 2021; Tang et al., 2022). This study hypothesized that the perception of social support mediates the relationship between negative life events and life satisfaction.

Sense of security not only refers to an individual’s premonition of physical and psychological dangers, but also includes a sense of strength or powerlessness when dealing with risks, mainly represented by interpersonal security and a sense of control (An et al., 2004). Maslow considered that sense of security was the feeling of individual happiness, trust, and love and being loved (Maslow et al., 1945). To provide just one theoretical angle, there is a growing literature on the relationship among negative life events and social relationship from the life history theory perspective (Reynolds and McCrea, 2016). Research of this tradition has found links between negative life events and social connections with family and friends (Zhu et al., 2018). Negative life events, as a key part of the latent construct of “uncertainty and unpredictability” were also linked to a “slow” life-history profile, which include relationship with parents and other social support and contact (Chang et al., 2019). Early experiences of negative life events might contribute to the ongoing “calibration” of “life-history strategy” in children and adolescents, affecting a range of psychological variables, including social investment in relationships with family and friends (Yang et al., 2022). These psychological strategies or “life-history profile” would have repercussions (e.g., estranged family and friends), which, in turn, contribute to an insecure, unpredictable schema of the world (Cabeza de Baca et al., 2016). Previous research has confirmed that negative life events in children are negatively correlated with the sense of security (Huang et al., 2016). Children with a low sense of security who experience negative life events may also experience negative emotions such as hostility and sadness, which results in distrust of the outside world and reduced life satisfaction (Mingming, 2012; Ting, 2020). Thus this study hypothesized that the sense of security mediates the relationship between negative life events and life satisfaction.

In addition, American scholar Bronfenbrenner once proposed in ecosystem theory that individuals and environment are nested in a system, and individual development is influenced by both internal and external factors (Bronfenbrenner, 1989). It can be inferred from the theory that left-behind children’s sense of security will be affected by both life events and social support. Some empirical research supports this. A recent empirical study found the sense of security mediates the relationship between negative life events and life satisfaction among migrant children. Jiang found that among a group of college students, a sense of security mediated the role between perceived social support and life satisfaction, which was due to individuals with highly perceived social support having a stronger sense of control when dealing with daily life events and interpersonal relationships (Jiang et al., 2019). The results show that having a higher sense of security and making better subjective evaluations of their life statuses lead to lower life satisfaction. Thus the study hypothesized that perceived social support and a sense of security act as a chain med1iator between them.

**Figure 1:**
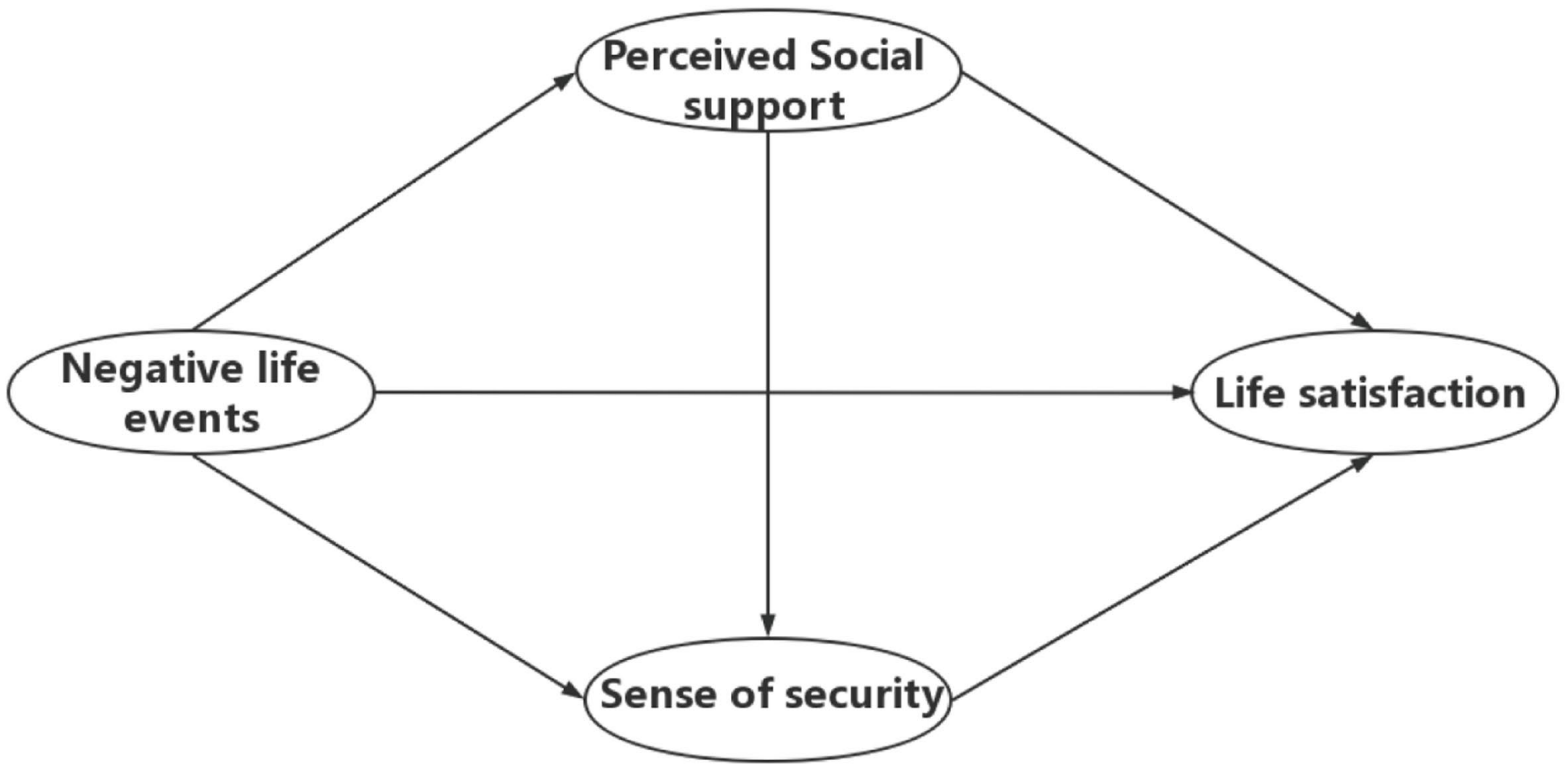
Hypothesized relationships among negative life events, perceived social support, sense of security, and life satisfaction.

Based on previous studies and theories, we aimed to determine the relationships among negative life events, perceived social support, a sense of security, and life satisfaction, and to construct a theoretical framework as detailed in Figure 1. This study collected data related to LBC in rural areas of Shandong province, China, and formulated the following hypotheses:

*Hypothesis 1*: Perceived social support mediates the relationship between negative life events and life satisfaction in LBC.

*Hypothesis 2*: Sense of security mediates the relationship between negative life events and life satisfaction in LBC.

## 2. Materials and methods

### 2.1. Study design and participants

Data were collected from Shandong province, China, which has one of largest numbers of LBC among provinces in China (Zhao et al., 2012; Deng et al., 2021). The province includes 17 cities and comprises about 15,000 LBC. The study was conducted from April to June 2020, and a multistep sampling process was used to recruit samples. First, according to geographical position and local economic conditions, 17 cities were randomly divided into 2 groups, and 1 city was selected from each group. Second, two counties were randomly drawn from each of the selected cities. Third, we used cluster sampling to randomly select 1 primary and 1 secondary school from 4 counties, including 20 classes and 5 grades (5th to 9th grades). The inclusion criteria were as follows: (1) participants and their guardians agreeing to participate, (2) one or both of their parents having left their respective city as migrant workers for more than 6 months, leaving them behind in the rural area, and (3) between the ages of 10 and 16 years. Exclusion criteria were as follows: (1) participants had history of mental health issues, and (2) participants who answered less than 50% of the questionnaires.

Researchers were familiar with and connected with the head teacher of each class, who assisted in obtaining verbal consent from the participants and their guardians. This study was approved by the Ethics Committee of Binzhou Medical University (approval number 2021-340). Participants took 20–30 min to complete each questionnaire in the classroom. The questionnaire was filled out by 290 participants, of which 281 LBC were identified as the final samples.

### 2.2. Measures

#### 2.2.1. Basic information

Basic information included gender, age, school stages, only-child, and types of being left, years of separation, time of living with parents, and minder.

#### 2.2.2. Adolescent self-rating life event checklist

Negative life events of LBC were assessed using the Chinese version of ASLEC. A score of between 1 and 6 was given according to the psychological feelings at the time of the event (e.g., “Long time away from the family can not be reunited,” “Disputes with your classmates or good friends”), with 1 indicating “normal feelings” and 6 indicating “extremely severe effect.” The higher the score indicated the greater total stress in the LBC. This scale has been widely used and validated in empirical studies in China (Liu et al., 1997a,b; Zheng et al., 2012). The scale contained 27 items, which were divided 6 dimensions, as follows: interpersonal relationship, study pressure, punishment, losing, health adaption, and others. The overall score of the subscales and scale were used as a single continuous variable. The Cronbach’s alpha for each dimension was 0.79, 0.8, 0.91, 0.81, 0.73, and 0.80 in the study. And the Cronbach’s alpha for ASLEC was 0.81.

#### 2.2.3. Perceived social support

In this study, the Chinese version of the Perceived Social Support Scale was used to assess the level of perceived social support of left-behind children [e.g., “I can share happiness and sadness with some people (teachers, relatives, classmates)”]. The scale was compiled by Zimet et al. (1990) and revised into Chinese version by Jiang (2001). And the scale was suitable for Chinese people (Gong Xun et al., 2022; Qing et al., 2022). This scale comprises 12 items and was scored on a 7-point Likert scale. And each item was graded from “strongly disagree” to “strongly agree.” The scale was distributed to 3 dimensions, as follows: support from family, from friends, and from others. The overall score of the full scale was used as a single continuous variable. This scale has been found to have good internal consistency, reliability, and validity. And this was also the case in the present study, with a Cronbach’s alpha of 0.88.

#### 2.2.4. Security questionnaire

We used the SQ to measure the security of LBC. The questionnaire was compiled by An et al. (2004). There were 16 items and 2 dimensions in the scale: interpersonal security and a sense of control. It was rated from 1 to 5, ranging from “completely fully compliance” to “completely out of line” (e.g., “I have never dared to speak out in my opinion”). These scales have been proved to be suitable for Chinese children (Wan et al., 2021). Psychometric property analysis indicated that the content and criterion-related validity scales achieved good levels in this stud. And Cronbach’s alpha values were 0.88 and 0.89 for interpersonal security and a definite sense of control, respectively. The overall score of the full scale was used as a single continuous variable, with a Cronbach’s alpha of 0.92.

#### 2.2.5. Life satisfaction

The Student’s Life Satisfaction Scale was compiled by Huebner, and has been adopted in several countries including the US, Spain, and South Korea. In 2004, Zhang translated this scale into Chinese (Zhang et al., 2004), which was regarded as the most suitable for Chinese populations (Xie et al., 2014). This scale contained 37 items and was scored on a 5-point Likert scale (e.g., “I have achieved the ideal results in my studies,” “Basically no one forced me to do things I do not like”). It was scored from 1 to 5, that is, from “indicating not at all” to “indicating certainly.” In the present study, the full scale was considered as a single continuous variable, which has good reliability in the study. The Cronbach’s alpha value was 0.81.

### 2.3. Data analysis

We used EpiData (version 3.11) for data input and SPSS (version 26.0) and Mplus (version 8.0) for data analysis, which was performed in three steps: (1) EpiData was used to set up and double-checked a database, (2) SPSS was used to describe the basic information of the participants, and (3) Mplus was used to analyze the fit of the hypothesized structural equation model. This assessed the indirect effects of negative life events on life satisfaction: (a) The indirect path *via* social support, (b) the indirect path *via* security, and (c) the indirect path *via* social support to security. In this study, Bootstrap = 2,000 was used for the test of mediation effect the estimation of confidence intervals. If 95% confidence intervals [CI] did not include 0, the indirect effect was considered significant. We also used other indicators with the following criteria to evaluate the goodness of fit for all models: CFI ≥0.90, *χ*^2^ < 3, *χ*^2^/df < 5; RMSEA ≤0.08, *p* < 0.00 and SRMR ≤0.05 (Hu and Bentler, 1999).

## 3. Results

### 3.1. Descriptive statistics

The study recruited 281 LBC with a mean age of 12.93 years. The proportion of LBC who attended junior high school was 67.26, 54.45% had fathers working outside the home, and 65.48% had grandparents caring for them (Table 1).

**Table 1:** Basic information of left-behind children (*N* = 281).

Projects		*N*	%
Gender	Male	154	54.80
	Female	127	45.20
School stages	Primary Schools	92	32.74
	Junior High School	189	67.26
Only-child	No	163	58.01
	Yes	118	41.99
Types of being left	Father working outside the home	153	54.45
	Mother working outside the home	59	21.00
	Both parents work outside the home	69	24.56
Years of separation	Less than 1 year	96	34.16
	1–2 years	70	24.91
	2–3 years	62	22.06
	More than 3 years	53	18.86
Time of living with parents	Within 15 days	74	26.33
	Within 30 days	92	32.74
	Within 2 months	79	28.11
	More than 2 months	36	12.81
Minder	Maternal grandparents	184	65.48
	Relatives	61	21.71
	Themselves	36	12.81

### 3.2. The correlation analysis

The total scores of the LBC on the scales for negative life events, perceived social support, sense of security, and life satisfaction were 68.87 ± 24.44 (Mean ± SD), 47.53 ± 17.73, 51.20 ± 12.21, and 118.20 ± 23.00. Perceived social support had a significant positive correlation with a sense of security and life satisfaction (*p* < 0.001), with correlation coefficients of 0.97 and 0.64, respectively. The sense of security had a significant positive correlation with life satisfaction (*p* < 0.001), with a correlation coefficient of 0.66 (Tables 2, 3).

**Table 2:** Correlations among negative life events, perceived social support, sense of security, and life satisfaction (*N* = 281).

	1	2	3	4	5	6	7	8	9	10	11	12	13	14	15	16	17	18	19	20	21
1. Interpersonal relationship	1																				
2. Study pressure	0.81^**^	1																			
3. Punishment	0.74^**^	0.83^**^	1																		
4. Losing	0.66^**^	0.67^**^	0.77^**^	1																	
5. Health adaption	0.70^**^	0.70^**^	0.69^**^	0.68^**^	1																
6. Others	0.75^**^	0.76^**^	0.86^**^	0.76^**^	0.77^**^	1															
7. Negative life event	0.87^**^	0.89^**^	0.94^**^	0.84^**^	0.82^**^	0.91^**^	1														
8. Family support	−0.23^**^	−0.30^**^	−0.34^**^	−0.31^**^	−0.33^**^	−0.35^**^	−0.35^**^	1													
9. Friend support	−0.26^**^	−0.31^**^	−0.34^**^	−0.32^**^	−0.32^**^	−0.33^**^	−0.35^**^	0.85^**^	1												
10. Others support	−0.25^**^	−0.31^**^	−0.35^**^	−0.033^**^	−0.34^**^	−0.35^**^	−0.36^**^	0.96^**^	0.96^**^	1											
11. Social support	−0.32^**^	−0.33^**^	−0.44^**^	−0.40^**^	−0.38^**^	−0.45^**^	−0.44^**^	0.32^**^	0.40^**^	0.37^**^	1										
12. Interpersonal security	−0.30^**^	−0.28^**^	−0.39^**^	−0.37^**^	−0.38^**^	−0.38^**^	−0.40^**^	0.29^**^	0.39^**^	0.35^**^	0.92^**^	1									
13. Sense of control	−0.31^**^	−0.30^**^	−0.44^**^	−0.40^**^	−0.41^**^	−0.44^**^	−0.43^**^	0.32^**^	0.40^**^	0.37^**^	0.90^**^	0.88^**^	1								
14. Sense of security	−0.32^**^	−0.31^**^	−0.44^**^	−0.40^**^	−0.40^**^	−0.44^**^	−0.44^**^	0.32^**^	0.41^**^	0.37^**^	0.97^**^	0.97^**^	0.96^**^	1							
15. Friendship satisfaction	−0.43^**^	−0.44^**^	−0.49^**^	−0.44^**^	−0.49^**^	−0.50^**^	−0.53^**^	0.44^**^	0.47^**^	0.47^**^	0.57^**^	0.55^**^	0.58^**^	0.55^**^	1						
16. Family satisfaction	−0.45^**^	−0.48^**^	−0.56^**^	−0.47^**^	−0.49^**^	−0.52^**^	−0.56^**^	0.44^**^	0.48^**^	0.48^**^	0.64^**^	0.63^**^	0.65^**^	0.66^**^	0.82^**^	1					
17. School satisfaction	−0.41^**^	−0.41^**^	−0.48^**^	−0.41^**^	−0.46^**^	−0.46^**^	−0.51^**^	0.37^**^	0.44^**^	0.42^**^	0.53^**^	0.56^**^	0.54^**^	0.56^**^	0.73^**^	0.77^**^	1				
18. Study satisfaction	−0.40^**^	−0.45^**^	−0.43^**^	−0.35^**^	−0.43^**^	−0.39^**^	−0.47^**^	0.36^**^	0.38^**^	0.38^**^	0.47^**^	0.49^**^	0.50^**^	0.50^**^	0.72^**^	0.67^**^	0.70^**^	1			
19. Freedom satisfaction	−0.47^**^	−0.53^**^	−0.55^**^	−0.48^**^	−0.52^**^	−0.51^**^	−0.58^**^	0.39^**^	0.47^**^	0.45^**^	0.61^**^	0.60^**^	0.60^**^	0.62^**^	0.78^**^	0.84^**^	0.74^**^	0.75^**^	1		
20. Environment satisfaction	−0.40^**^	−0.41^**^	−0.45^**^	−0.35^**^	−0.43^**^	−0.48^**^	−0.48^**^	0.33^**^	0.39^**^	0.37^**^	0.54^**^	0.54^**^	0.53^**^	0.56^**^	0.68^**^	0.75^**^	0.76^**^	0.58^**^	0.73^**^	1	
21. Life satisfaction	−0.48^**^	−0.52^**^	−0.57^**^	−0.48^**^	−0.53^**^	−0.54^**^	−0.59^**^	0.45^**^	0.50^**^	0.49^**^	0.64^**^	0.63^**^	0.64^**^	0.66^**^	0.90^**^	0.93^**^	0.88^**^	0.82^**^	0.92^**^	0.83^**^	1
Mean	12.71	13.28	17.59	7.69	7.84	10.08	68.87	16.00	16.25	15.26	47.53	25.86	25.35	51.20	23.21	21.99	20.42	20.12	15.27	17.23	118.20
SD	4.46	4.84	7.65	3.36	3.00	4.17	24.44	5.89	6.36	6.09	17.73	6.43	6.27	12.21	4.41	6.33	4.08	3.72	4.38	3.02	23.00

**P < 0.01.

**Table 3:** Spearman’s correlation matrix of the study variables (*N* = 281).

	Only-child	Time of living with parents	Minder
Negative life event	0.083	−0.116	0.035
Social support	−0.163**	0.194**	−0.165**
Sense of security	0.059	0.08	−0.026
Life satisfaction	−0.131*	0.187**	−0.170**

*P < 0.05, **P < 0.01.

### 3.3. SEM model testing

**Figure 2:**
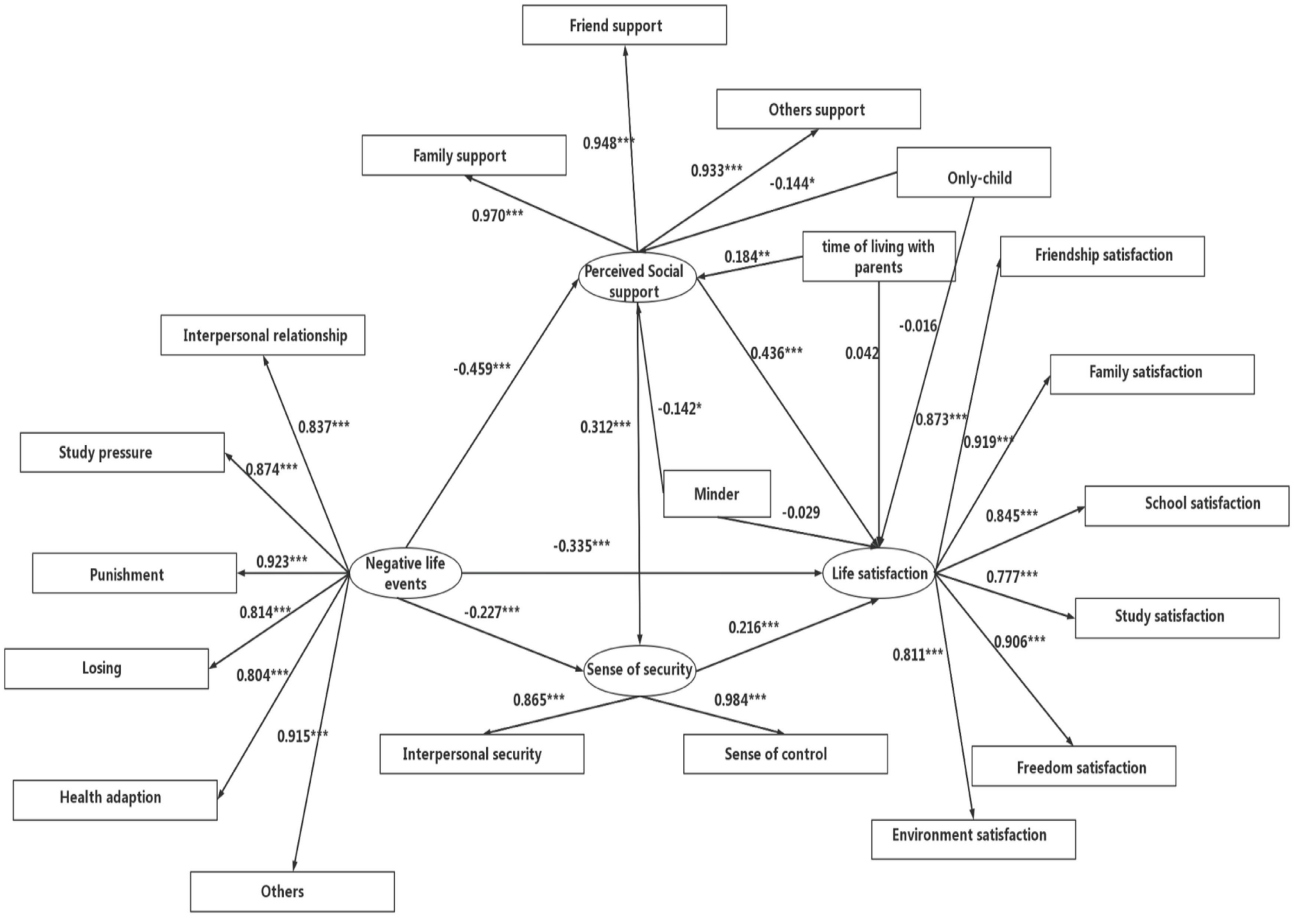
Chain-mediating effect of perceived social support and sense of security (all values are normalized). ^*^p < 0.05, ^**^p < 0.01, ^***^p < 0.001.

After controlling for only-child, time of living with parent and minder, path diagrams for the four components of perceived social support, sense of security, negative life events, and life satisfaction are shown in Figure 2. Perceived social support and sense of security played partially mediating roles in the effect of negative life events on life satisfaction, with model fit indices of CFI = 0.95, *χ*^2^ = 403.10, df = 161, *χ*^2^/df = 2.50, RMSEA = 0.07, *p* < 0.001, and SRMR = 0.04.

The direct effect value of negative life events on life satisfaction was −0.335, with an effect share of 53.86%. The mediating effect between negative life events and life satisfaction consisted of indirect effects along three pathways: the first was generated by negative life events → perceived social support → life satisfaction, with an effect value of −0.200 and an effect share of 32.15%; the second was generated by negative life events → sense of security → life satisfaction, with an effect value of −0.049 and an effect share of 7.88%; and the third was generated by negative life events → perceived social support → sense of security → life satisfaction, with an effect value of −0.031 and an effect share of 4.98% (Table 4).

**Table 4:** Chain-mediating effects of perceived social support and sense of security on negative life events and life satisfaction (*N* = 281).

Paths	Estimate	S.E	Est/S.E	*P*	95% CI		Percentage of effect
					Lower	Upper	
Total effect	−0.622	0.044	−13.946	<0.001	−0.699	−0.525	100.00
Direct effects	−0.335	0.060	−5.551	<0.001	−0.456	−0.208	53.86
Indirect effects 1	−0.200	0.043	−4.693	<0.001	−0.290	−0.122	32.15
Indirect effect 2	−0.049	0.022	−2.190	0.033	−0.099	−0.011	7.88
Indirect effects 3	−0.031	0.012	−2.642	0.012	−0.056	−0.011	4.98
Total indirect effect	−0.287	0.050	−5.730	<0.001	−0.391	−0.191	46.14

Indirect effect 1 (Negative life events → Perceived social support → Life satisfaction), indirect effect 2 (Negative life events → Sense of security → Life satisfaction), Indirect effect 3 (Negative life events → Perceived social support → Sense of security → Life satisfaction).

## 4. Discussion

This study constructed a chain-mediating model to explore the effects of negative life events, perceived social support, and a sense of security on life satisfaction. It was found that perceived social support and a sense of security influence the relationship between negative life events and life satisfaction, which contributes to a comprehensive understanding of the mechanisms that influence life satisfaction. The findings of the study have important theoretical value for reducing the emergence of psychological problems among LBC, as well as for enhancing their life satisfaction (Table 4).

Negative life events were found to be negatively correlated with perceived social support, which may be related to the tendency of LBC to avoid coping styles when encountering negative life events such as interpersonal conflicts, academic stress, and violence in school (Gao B. et al., 2022). In addition, Tang et al. have also shown that negative life events will affect an individual’s emotional experience, which makes the individual unable to feel support from family and friends, and eventually have more negative effects on the individual (Tang et al., 2022). There was also a negative correlation between negative life events and a sense of security, which may be due to the insecurity felt by LBC in their home environment and discordant school environment when they suffer from other negative life events such as punishment from parents and teachers as well as academic stress (Gao C. et al., 2022). When they are then subjected to these other negative life events, they may feel insecure in their home environment and discordant school environment, thus reducing their sense of security. There was also a negative correlation between negative life events and life satisfaction, which may be related to LBC experiencing negative life events often experiencing uncomfortable emotions such as psychological distress, social anxiety, and depression, thus reducing their life satisfaction. This study also found positive correlations among perceived social support, a sense of security, and life satisfaction. Perceived social support is an important psychosocial resource that can help patients feel the care and support of the outside world and increase their sense of control and belonging (Chen et al., 2021), which can improve their sense of security. Perceived social support and a sense of security can also lead to a sense of hope and meaning in life (Fu et al., 2022; Gorgol et al., 2022), which can increase life satisfaction. This study further validated the constructed structural equation model based on the above findings.

This study found that the perceived social support of LBC mediated the relationship between negative life events and life satisfaction, which confirmed Hypothesis 1. This may be related to LBC often being blamed by parents and criticized by teachers for a negative life event (Li et al., 2021), so they cannot feel the love and support of those around them when these events occur. It might also be related to LBC not experiencing social support, resulting in them considering themselves unworthy of care, having no sense of purpose, thinking that life is meaningless (Jekauc et al., 2019; Zhang L. Y. C. et al., 2022), and losing hope in life, resulting in reduced life satisfaction. The above findings suggest that parents and teachers need to promptly and comprehensively understand the causes and processes of negative life events in LBC, provide adequate support and assistance, choose appropriate educational approaches to guide LBC to resolve the events they experience in their lives, and strengthen the relationship with them. Parents and teachers should also increase the frequency of communication with LBC, and incorporate life education into their conversations to help them establish a better outlook on life and values.

This study also found that the sense of security among LBC played a mediating role between negative life events and life satisfaction, which was consistent with the findings of Rowe et al. (2013). LBC have a sense of powerlessness in coping with negative life events and may adopt a negative attribution bias (Shi, 2022). They perceive negative life events as uncontrollable and anticipate them. Negative life events will threaten their physical and mental health (Guo et al., 2005), which will cause them to have negative emotions (such as hostility and resentment toward the surrounding environment) while lacking a sense of security (Zhang H. et al., 2022). Sense of security is the foundation of an individual’s mental health. Low levels of security not only prevent individuals from correctly evaluating negative life events, but also fail to adopt positive and effective coping behavior (Widmer et al., 2023). Individuals will have a variety of negative emotions such as anxiety and fear due to fear of the recurrence of negative life events, which will eventually affect the life satisfaction of LBC (Jia et al., 2021).

In addition, the relationship between negative life events and life satisfaction was found to be mediated by a chain between perceived social support and a sense of security. Negative life events reduce the level of perceived social support in LBC, which makes them prone to other psychological problems (Hassija and Gray, 2012; Chang et al., 2019). This can lead to a feeling of discomfort and dissatisfaction in their lives, as they believe that others are isolating and discriminating against them and that they are not trusted and accepted by others (Chen and Chang, 2012). The main effects model states that perceiving low social support affects the psychological state of individuals, resulting in anxiety, other psychological problems and the fears of losing existing interpersonal relationships and feelings of insecurity (Peirce et al., 1996; Yang, 2022). This ultimately leads to low levels of subjective well-being and satisfaction with family, friendships, and school. For this reason, social support sources for LBC should be increased, using methods such as enriching school activities and expanding peer networks to increase peer support; promoting local employment for farmers, which would solve the problem of long-term separation between parents and children to increase family support; and caring members of society setting up “love huts” and carrying out psychological counseling activities to strengthen other support sources. Support sources should be strictly implemented as above to improve the sense of security of LBC. In short, the impacts of negative life events on life satisfaction can be reduced by increasing the levels of social support and sense of security perceived by LBC.

Unlike previous studies, this study not only explored the factors that influence life satisfaction among LBC but also revealed the roles of perceived social support and sense of security in the relationship between negative life events and life satisfaction. However, this study was subject to the following limitations: First, due to research funding restrictions, this study only investigated the mechanism of the role between negative life events and life satisfaction among LBC in rural Shandong, and its findings might not be applicable to other regions because of differences in regional policies and economic conditions. Second, this study only revealed the mediating roles of perceived social support and a sense of security on the relationship between negative life events and life satisfaction, and other pathways between negative life events and life satisfaction still need to be investigated in the future.

## 5. Conclusion

This study explored the relationship between negative life events, perceived social support, sense of security and life satisfaction of LBC. There was a significant negative correlation between negative life events and life satisfaction among LBC. In the meantime, negative life events could indirectly predict life satisfaction among LBC through the chain-mediating effects of perceived social support and sense of security. The two mediators, i.e., perceived social support and a sense of security, provide the targets for enhancing life satisfaction among LBC. The results also indicated that schools, patients of LBC, and society could develop interventions that enhance LBC’s perceived social support and a sense of security that in turn would improve their life satisfaction.

## Data availability statement

The raw data supporting the conclusions of this article will be made available by the authors, without undue reservation.

## Ethics statement

This study was approved by the Ethics Committee of Binzhou Medical University (approval number 2021-340). Verbal Consent for participation was obtained from the participants’ legal guardians/next of kin.

## Author contributions

NL was involved in the data analysis, writing the original draft preparation, and constructing the model. XL contributed to the study design and modify the original draft preparation. XD was involved in data collection and the writing of the original draft preparation. HL was involved in study design and data collection. XZ was involved in study design, data analysis, and modify the original draft preparation. All authors contributed to the article and approved the submitted version.

## Funding

This project was completed with funding from the Soft Science Research Program of Shandong Province (2015RKB14072) and Binzhou Medical University Social Science Development Foundation (20SKSY04).

## Conflict of interest

The authors declare that the research was conducted in the absence of any commercial or financial relationships that could be construed as a potential conflict of interest.

## Publisher’s note

All claims expressed in this article are solely those of the authors and do not necessarily represent those of their affiliated organizations, or those of the publisher, the editors and the reviewers. Any product that may be evaluated in this article, or claim that may be made by its manufacturer, is not guaranteed or endorsed by the publisher.
